# Vertical transmission, infection dynamics and risk factors for the occurrence of tick fever agents in beef cattle from a tropical region

**DOI:** 10.1007/s11250-026-05341-x

**Published:** 2026-09-21

**Authors:** Lidia Mendes de Aquino, Igor Maciel Lopes de Morais, Vanessa Ferreira Salvador, Luccas Lourenzzo Lima Lins Leal, Warley Vieira de Freitas Paula, Gabriel Cândido dos Santos, Luiz Felipe Monteiro Couto, Dina Maria Beltran Zapa, Luciana Maffini Heller, Marcus Antonio Martins Buso, Evandro Davanco Ferreira de Souza, Felipe da Silva Krawczak, Lorena Lopes Ferreira, Camila Valgas Bastos, Emmanuel Arnold, Rosangela Zacarias Machado, Vando Edésio Soares, Welber Daniel Zanetti Lopes

**Affiliations:** 1 Escola de Veterinária e Zootecnia, Universidade Federal de Goiás, Goiânia, Goiás Brazil; 2 Departamento de Medicina Veterinária Preventiva, Escola de Veterinária, Universidade Federal de Minas Gerais, Belo Horizonte, Minas Gerais Brazil; 3 Departamento de Patologia Animal, Faculdade de Ciências Agrárias e Veterinárias, Universidade Estadual Paulista, Jaboticabal, São Paulo, Brazil; 4 Universidade Brasil, Descalvado, São Paulo, Brazil; 5 Departamento de Biociências e Tecnologia, Instituto de Patologia Tropical e Saúde Pública, Universidade Federal de Goiás, Goiânia, Goiás Brazil

**Keywords:** Anaplasmosis, Babesiosis, Cattle tick, Enzootic stability

## Abstract

This study investigated the vertical transmission, infection dynamics, and risk factors associated with tick fever agents in beef calves raised in a tropical region over a two-year period. A total of 98 cow–calf pairs were monitored from birth until slaughter (males) or first calving (females). Blood samples were analyzed by qPCR and iELISA for *Anaplasma marginale*, *Babesia bovis*, and *Babesia bigemina*. Vertical transmission was detected only for *A. marginale* (6.5%). At 20 days of age, qPCR detected *A. marginale* DNA in 85% of calves and *Babesia* spp. DNA in 60–80%, indicating early exposure and active infection. Tick burden was the only variable independently associated with both blood smear positivity and bacteremia, providing observational evidence of a potential epidemiological role of *R. microplus* in *A. marginale* infection under field conditions. Low *Babesia* parasitemia precluded comparative analyses between sexes. Serological prevalence exceeded 75%, indicating widespread exposure within the herd. Nevertheless, clinical cases of anaplasmosis occurred in 20-day-old calves, 8–9-month-old steers, and 13–14-month-old heifers, suggesting that seropositivity alone was not sufficient to prevent clinical disease. These findings reveal significant shifts in the epidemiology of tick fever under tropical conditions, characterized by early infection, widespread pathogen exposure, and the persistence of clinical disease, emphasizing the need to reassess current control strategies for tick fever in beef herds.

## Introduction

Tick fever (TF) is a disease complex caused by infections with *Babesia bovis*, *Babesia bigemina*, and *Anaplasma marginale*. It is one of the major constraints on beef and dairy cattle production in tropical and subtropical regions. Clinically, the disease is characterized by fever, anemia, jaundice, and, in severe cases, high mortality rates. These outcomes result in substantial economic losses due to reduced productivity, increased treatment costs, and animal deaths (De Moraes et al. 2024). In cattle, *A. marginale* is primarily transmitted by the cattle tick *Rhipicephalus microplus* (Heller et al. 2026); however, mechanical transmission through hematophagous flies, contaminated needles, and transplacental transfer may also occur. Similarly, babesiosis is mainly spread by *R. microplus*, with occasional cases of vertical transmission reported (Alonso et al. 1992; Kessler et al., 2001; Reinbold et al. 2010; Costa et al. 2016).

Recent studies have reported an increasing frequency of outbreaks caused by TF agents in both beef and dairy cattle raised in tropical regions. In some of these cases, mortality rates may reach up to 20% of the herd (Machado et al. 2015; Leal et al. 2024). These findings highlight that, although TF is a well-known challenge in cattle production, the TF agents continue to cause significant harm to the herd. Part of this issue may be attributed to changes in management systems, combined with the use of genetically superior animals selected for higher productivity (De Moraes et al. 2024). In practice, the combination of these factors may be contributing to the increased frequency of clinical cases and mortality events currently being observed (Cavalcante et al. 2025).

Therefore, field-based studies that investigate the infection dynamics, clinical presentations, and potential risk factors associated with TF agents are essential. Such research not only provides insights into the current epidemiological patterns of infection but also supports the improved training of veterinarians and farmers, with the aim of minimizing the losses caused by ongoing TF outbreaks. This knowledge gap justified the development of the present study. The aim of the present study was to evaluate, over a two-year period, the vertical transmission, infection dynamics, and risk factors associated with the occurrence of TF agents in beef cattle raised in a tropical region.

## Materials and methods

### Ethics committee

This project was submitted to and approved, protocol no. 062/21, by the Ethics Committee for the Use of Animals (CEUA) of the Federal University of Goiás, following the ethical principles in animal experimentation of the National Council for the Control of Animal Experimentation (CONCEA).

### Study site and animal management

The study was conducted at Fazenda Reunidas Baumgart, located in the municipality of Rio Verde, Goiás, in the Central-West region of Brazil, and lasted for 26 months, from September 2020 to November 2022. The farm operates a full-cycle beef cattle production system under semi-intensive and intensive management. The regional climate is classified as tropical semi-humid, with two well-defined seasons: a dry season (May to September) and a rainy season (October to April), with average temperatures ranging from 20 °C to 35 °C (INMET, 2024).

**Fig. 1:**
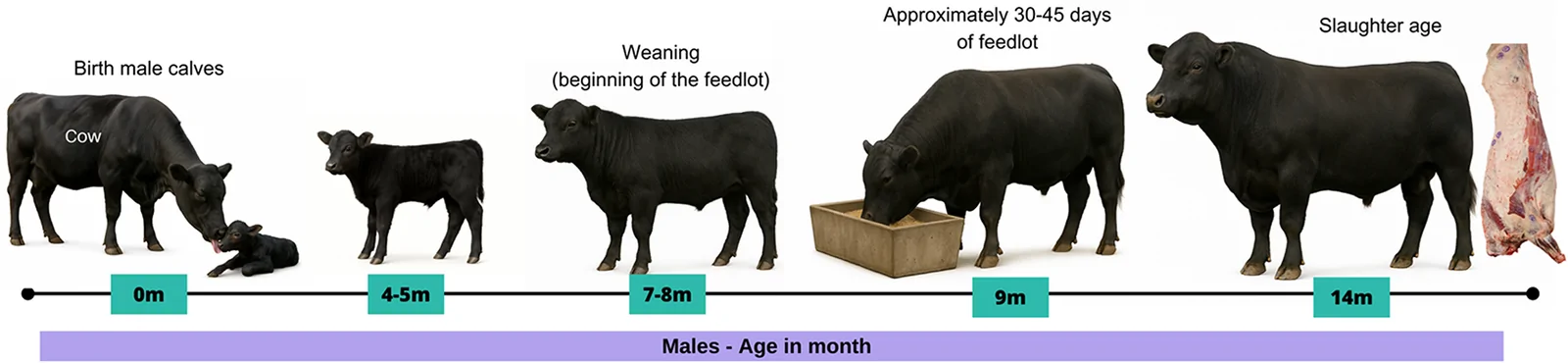
Experimental design adopted to the males calves birth until slaughter during the study

**Fig. 2:**
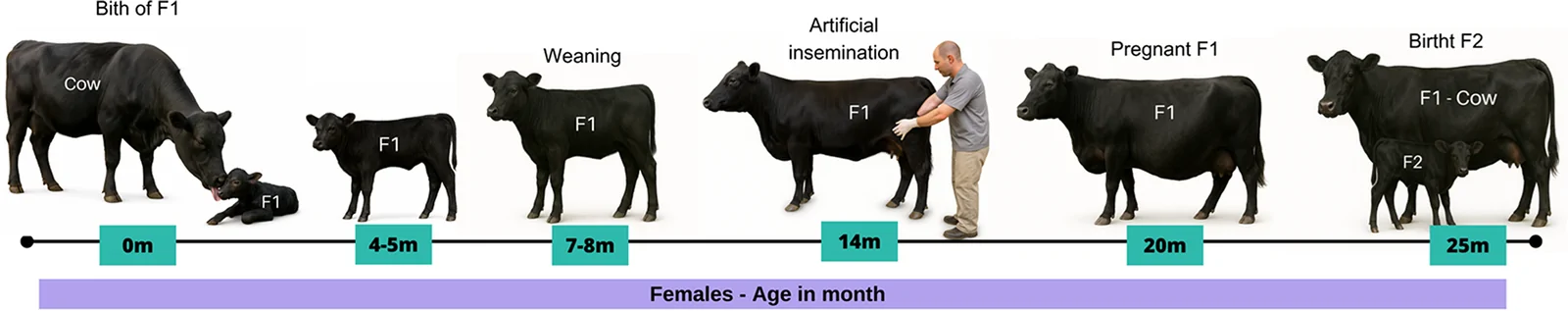
Experimental design adopted to the females’ calves’ birth until their partum during the study

A total of 98 cow–calf pairs were enrolled on the day of calving. The cows were evaluated only at parturition. Among the offspring, 52 were female and 46 were male. The calves were tricrosses obtained by inseminating F1 dams (½ Angus × ½ Nellore) with semen from Brangus, Braford, or Charolais bulls. All calves were monitored from birth to 20 days of age. Thereafter, 10 males were randomly selected for longitudinal follow-up until slaughter (approximately 14 months of age; Fig. 1), whereas 10 females were followed until their first calving, at approximately 25 months of age (Fig. 2).

Weaning occurred when the calves reached an average age of seven to eight months. Male calves were then moved to a feedlot, where they remained until slaughter. Female calves were kept on pasture throughout the entire study and were subjected to a fixed-time artificial insemination (FTAI) protocol at approximately 14 to 15 months of age.

During the suckling period, calves received maternal milk, proteinated creep-feed containing lasalocid, ground corn, soybean meal, and urea, with *ad libitum* access to water. After weaning, males were fed a feedlot diet consisting of corn silage, soybean meal, sugarcane bagasse, ground corn, urea, limestone, and a monensin-containing supplement until slaughter. Females remained on pasture and received a diet based on ground corn, soybean meal, urea, mineral salt, and a supplement formulated for replacement heifers until the end of the study.

### Evaluation of passive immunity transfer to calves

Three days after birth, total plasma protein (TPP) concentration was measured in plasma obtained from centrifuged blood samples using a VODEX optical refractometer (REFD model). According to McGuirk (2003), TPP concentrations > 5.5 g/dL were considered indicative of adequate passive transfer of maternal immunity. This evaluation was performed in all newborn calves to assess colostrum intake and the efficiency of passive transfer.

Total plasma protein (TPP) concentration was determined three days after birth and subsequently at 2, 4, 6, 8, 9, 14 (last evaluation for males), 21, 22, and 25 months of age using a veterinary refractometer (RZ200; automatic temperature compensation [ATC]; range: 0–14 g/dL), according to Schalm et al. (1975). All calves were evaluated until weaning, after which the longitudinal follow-up continued with the randomly selected subcohorts of 10 males and 10 females, as previously described.

### Detection of tick fever agents by molecular and serological methods, and evaluation of enzootic stability

Regarding molecular analysis by quantitative PCR (qPCR), blood samples were collected from all 98 cows and from all F1 calves at birth (within 6 h of delivery), 7 days, and 20 days of age to evaluate the vertical transmission of tick fever agents. Thereafter, the randomly selected subcohorts of 10 males and 10 females were sampled at 2, 4, 6, 8, 9, 14 (last evaluation for males), 21, 22, and 25 months of age. In addition, samples from 10 F2 calves were analyzed by qPCR at birth.

For each of these blood samples, 300 µL were subjected to DNA extraction using the Wizard^®^ Genomic DNA Purification kit (Promega, Madison, WI, USA), following the manufacturer’s instructions. The extracted DNA was analyzed using in three distinct qPCR assays, each targeting one of the following pathogens: *A. marginale*, *B. bovis* and *B. bigemina*. The qPCR assay targeted a fragment of the major surface protein 1b (*msp1b*) gene of *A. marginale* (Carelli et al. 2007), and the mitochondrial cytochrome B (*cytB*) gene of *B. bovis* and *B. bigemina*, as described by Criado-Forneli et al. (2009), with modifications to the quenchers (Table 1). Each qPCR run included a negative control (PCR-grade water, Sigma-Aldrich, St. Louis, MO, USA) and a pathogen-specific positive control (DNA from *A. marginale*, *B. bovis*, or *B. bigemina*).

**Table 1:** Oligonucleotides (primers and probes) used in the present study

Target gene / Specificity	Primer/probe: Sequence 5′–3′	Amplicon size (bp)	Reference
**qPCR**			
*msp 1b* / *A. marginale*	AM-For: TTGGCAAGGCAGCAGCTT AM-Rer: TTCCGCGAGCATGTGCAT AM-Pb: 5’ 6FAM-TCGGTCTAACATCTCCAGGCTTTCAT-3’ QSY	95 bp	Carelli et al. 2007
*cytB /* *B. bovis*	BOT-F: TGTTCCTGGAAGCGTTGATTC BOT-R: CCAACCCATATTGACTTCAGC BOT-P: 5’ VIC-TGAATGTGTAATTAGAGTGC-3’ MGB -NFQ	95 bp	Criado-Fornelio et al. 2009
*cytB* / *B. bigemina*	BIT-F: TTATGTTCCAGGAGATGTTGA BIT-R: CCCAACCCATATTAACCTCAGT BIT-P: 5’ FAM-CGAATGTGTTATCAGAGTAT- 3’ MGB -NFQ	95 bp	Criado-Fornelio et al. 2009
**PCR**			
*cytB* / Mamífero	L14841-F: CCATCCAACATCTCAGCATGATGAAA H15149-R: GCCCCTCAGAATGATATTTGTCCTCA	359 pb	Kocher et al. 1989

All qPCR assays were performed using TaqMan^®^ Environmental Master Mix 2.0 (Applied Biosystems^®^, Foster City, CA, USA) according to the manufacturer’s recommendations, in a StepOnePlus™ Real-Time PCR System thermal cycler (Applied Biosystems^®^, Foster City, CA, USA). Thermal cycling conditions included an initial DNA polymerase activation at 95 °C for 10 min, followed by 40 cycles of denaturation at 95 °C for 15 s and annealing-extension at 60 °C for 1 min. For all assays, lower CT indicated a higher quantity of target DNA. Samples with CT values > 35 were considered negative.

Negative samples were further tested using a conventional PCR (cPCR) protocol targeting a 359 bp fragment of the mammalian mitochondrial cytochrome B (*cytB*) protein gene Kocher et al. (1989) to validate the DNA extraction procedure. Samples that failed to amplify in this assay were excluded from further analysis. PCR products were stained with SYBR Safe (Invitrogen, Thermo Fisher Scientific, Waltham, MA, USA) according to the manufacturer’s recommendations and visualized on 1.5% agarose gel electrophoresis under an ultraviolet transilluminator.

Serological evaluation was performed by indirect ELISA (iELISA) in all cows (*n* = 98) and F1 calves. Calves were sampled at 20 days of age and subsequently at 2, 4, 6, 8, 9, and 14 months of age (last evaluation for males), with an additional sampling at 25 months for the female subcohort. IgG antibodies against *A. marginale*, *B. bovis*, and *B. bigemina* were detected using the iELISA protocols described by Machado et al. (1997) and Andrade et al. (2004). The cut-off values for *A. marginale*, *B. bigemina*, and *B. bovis* were 0.329, 0.410, and 0.373, respectively.

According to Mahoney and Roos (1972) and Lagranha et al. (2024), a herd is considered enzootically stable for *Babesia* spp. and *A. marginale* if more than 75% of the animals test positive for these pathogens by serological methods at or before nine months of age. Therefore, in this study, enzootic stability for each of the three tick fever agents was defined as a ≥ 75% positivity rate in a group at or before nine months age, based on serological results. The qPCR results were used only as a complementary analysis.

### Blood collection for blood smear and packed cell volume (PCV) analysis

Blood samples were collected from all cows at calving and from their respective F1 calves at birth, 7, and 20 days of age. Thereafter, calves were sampled at 2, 4, 6, 8, 9, and 14 months of age (last evaluation for males), with additional collections at 21, 22, and 25 months for the female subcohort. All calves were evaluated until weaning, after which the randomly selected group of 10 females continued to be monitored until the end of the study.

The parasitological diagnosis of the TF agents was done using blood collected from the tip of the animals’ tails, adding Giemsa to the blood smears which were subsequently examined under an optical microscope (1000x magnification). The percentage of bacteremia for *A. marginale* or parasitemia for *B. bovis* and *B. bigemina* was calculated following the method described by IICA (1984) and Coetzee et al. (2006).

For packed cell volume (PCV) measurement, approximately 4 mL of blood was collected from the coccygea vein of each animal into tubes containing the anticoagulant K_2_EDTA (BD^®^). The PCV was determined using the microhematocrit method, as described by Weiss and Wardrop (2011). In the laboratory, the blood samples were homogenized and transferred to capillary tubes (microhematocrit tubes), filling approximately 75% of the tube. The tubes were then centrifuged in a microcentrifuge at 13,000 × g for five minutes. Following centrifugation, PCV values were read using a standard microhematocrit reader card (Gomes et al. 2007). PCV values ≤ 24% or ≥ 46% were considered outside the normal physiological range, according to Feldman et al. (2000).

### Observation of hematophagous flies, tick counts, and acaricide treatments

The presence of hematophagous flies was monitored throughout the study period. From birth until 20 days of age, calves were inspected daily during routine clinical evaluations. Thereafter, fly assessments were performed concurrently with the scheduled tick counts. During each observation, the presence or absence of *Haematobia irritans*, *Stomoxys calcitrans*, and Tabanidae on each animal was recorded. First the presence or absence of fly was recorded based on the observation of defensive behaviors (leg and/or head movements), concomitantly with the direct visualization of flies on the animals, and which fly it was. Because the objective was to investigate the potential epidemiological association between hematophagous flies and *Anaplasma marginale* infection, fly occurrence was recorded as a binary variable (presence/absence) rather than by abundance.

Tick infestation was assessed by counting engorged *Rhipicephalus microplus* females (4.5–8.0 mm in length) on the left side of each animal, without doubling the count, according to Wharton and Utech (1970). Tick counts were performed at birth, 7 and 20 days of age, and subsequently at 2, 4, 6, 8, 9, and 14 months of age (last evaluation for males), with additional evaluations at 21, 22, and 24 months for the female subcohort. All calves were evaluated until weaning, after which the randomly selected females continued to be monitored until the end of the study. Acaricide treatments followed the farm’s routine management protocol using a formulation containing 1.25 mg/kg fipronil and 2.5 mg/kg fluazuron. Whenever treatment was administered, all animals were treated simultaneously and the treatment date was recorded.

### Curative and preventive treatments against tick fever pathogens

To ensure animal welfare throughout the experimental period, any animal showing clinical signs consistent with tick fever was promptly treated with a curative protocol. This included diminazene aceturate at 3.5 mg/kg (Ganaseg^®^ – Elanco Animal Health; Papich 2021) combined with enrofloxacin at 7.5 mg/kg administered intramuscularly (Knetomax^®^ – Elanco Animal Health; Facury-Filho et al. 2012). Each case was recorded with the animal’s identification number and the treatment date. Additionally, a blood smear was performed at the time of treatment to microscopically confirm the presence and identify the specific tick fever agent involved.

In cases where more than 4% of the calves (i.e., ≥ 4 animals) exhibited clinical signs of tick fever until 20 days of age, the entire calf cohort received a preventive treatment consisting of a single dose of imidocarb dipropionate at 1.2 mg/kg (Imizol^®^ – MSD Animal Health). This measure was based on the farm’s history of early outbreaks of tick fever (Leal et al. 2024).

### Statistical analysis

The serological data did not meet the assumptions of normality, homogeneity of variances, independence of residuals, or randomness, even after log (count + 1) transformation. Therefore, comparisons between experimental groups (males and females) were performed using the Kruskal–Wallis non-parametric test.

Longitudinal data were summarized according to age and sex. At each sampling time, the number of animals evaluated, blood smear positivity, mean tick count, and mean *A. marginale* bacteremia were calculated. Wilson 95% confidence intervals were estimated for blood smear positivity, whereas 95% confidence intervals based on the normal approximation were calculated for continuous outcomes.

Exploratory multivariable regression models were fitted using individual-level observations during the experimental period. Blood smear positivity was analyzed using multivariable logistic regression, whereas bacteremia was analyzed using multivariable linear regression. Bacteremia was evaluated both in the complete dataset and in a separate analysis restricted to blood smear-positive observations, allowing factors associated with the occurrence of infection to be distinguished from those associated with the intensity of bacteremia. The models included categorical sex, log-transformed tick count [log10(1 + tick count)], therapeutic treatment for bovine tick fever, packed cell volume (PCV), total plasma protein (TPP), and hematophagous flies concentration as explanatory variables. Robust standard errors clustered by animal identification were used to account for the correlation among repeated observations obtained from the same animal. Results were expressed as odds ratios (ORs) or regression coefficients (β), together with their respective 95% confidence intervals (95% CIs) and *P*-values. Statistical significance was defined as *P* < 0.05. All statistical analyses were performed using the Statistical Analysis System (SAS, 2016), version 9.4.

The low parasitemia by *B. bovis* and *B. bigemina* in the calves made it impossible to perform statistical analysis for these agent.

## Results

### Vertical transmission and infection dynamics of tick fever agents in F1 calves assessed by qPCR

The results of qPCR are shown in Tables 2 and 3. In the dams of the male calves, 91.3% (42/46), 36.95% (17/46), and 8.6% (4/46) were infected with *A. marginale*, *B. bovis*, and *B. bigemina*, respectively. Regarding the calves, at birth (vertical transmission), three F1 male calves (6.5% − 3/46) tested positive for *A. marginale* by qPCR. Only those born with *A. marginale* remained infected with this rickettsia at seven days of age (3/46–6.5%). No F1 male calves tested positive for *B. bovis* or *B. bigemina* from birth until 7 days of age. By 20 days of age, 84.8% (39/46), 80.4% (37/46), and 78.3% (36/46) of the calves were infected with *A. marginale*, *B. bovis*, and *B. bigemina*, respectively (Table 2). Subsequently, specifically for *A. marginale*, the occurrence was 50% (5/10) and 70% (7/10) at 2 and 4 months of age, remained between 80% (8/10) and 90% (9/10) from 6 to 9 months, and was 60% (6/10) at 14 months of age, when the animals were slaughtered (Table 2).

**Table 2:** Detection of *Anaplasma marginale*,* Babesia bigemina and Babesia bovis* by qPCR in beef cows and male calves since the birth until 14 months (slaughter)

Age in day (d) or month (m)	Tick fever agent and number of animals positive by qPCR (frequency %)				
	*Anaplasma marginale*		*Babesia bovis*		*Babesia bigemina*
Cow	42/46 (91.3)		17/46 (36.95)		4/46 (8.6)
0 (Birth F1)	3/46 (6.5)		0/46 (0.0)		0/46 (0.0)
7d	3/46 (6.5)		0/46 (0.0)		0/46 (0.0)
20d *	39/46 (84.8)		37/46 (80.4)		36/46 (78.3)
2 m *	5/10 (50.0)		0/10 (0.0)		0/10 (0.0)
4 m	7/10 (70.0)		3/10 (30.0)		0/10 (0.0)
6 m *	9/10 (90.0)		8/10 (80.0)		8/10 (80.0)
8 m (weaning)	9/10 (90.0)		5/10 (50.0)		7/10 (70.0)
9 m (feedlot) *	8/10 (80.0)		6/10 (60.0)		6/10 (60.0)
14 m (slaughter)	6/10 (60.0)		5/10 (50.0)		0/10 (0.0)

* Some animals received curative protocol: diminazene aceturate at 3.5 mg/kg and enrofloxacin at 7.5 mg/kg

**Table 3:** Detection of *Anaplasma marginale*,* Babesia bigemina and Babesia bovis* by qPCR in beef cows and female calves since the birth until 25 months (F1 calving F2)

Age in days (d) or month (m)	Tick fever agent and number of animals positive for tick fever agents by qPCR (frequency %)				
	*Anaplasma marginale*		*Babesia bovis*		*Babesia bigemina*
Cow	48/52 (92.3)		21/52 (40.38)		3/52 (5.7)
0 (Birth F1)	0/52 (0.0)		0/52 (0.0)		0/52 (0.0)
7d	0/52 (0.0)		0/52 (0.0)		0/52 (0.0)
20d *	45/52 (86.5)		17/52 (32.7)		27/52 (51.9)
2 m *	4/10 (40.0)		1/10 (10.0)		0/10 (0.0)
4 m *	3/10 (30.0)		4/10 (40.0)		1/10 (10.0)
6 m *	6/10 (60.0)		6/10 (60.0)		6/10 (60.0)
8 m	7/10 (70.0)		7/10 (70.0)		8/10 (80.0)
9 m	8/10 (80.0)		4/10 (40.0)		5/10 (50.0)
14 m (reproduction) *	8/10 (80.0)		10/10 (100.0)		3/10 (30.0)
21 m	5/10 (50.0)		8/10 (80.0)		3/10 (30.0)
22 m	7/10 (70.0)		4/10 (40.0)		6/10 (60.0)
25 m (Cow calving F2)	6/10 (60.0)		5/10 (50.0)		5/10 (50.0)
Calving F2	0/10 (0.0)		0/10 (0.0)		0/10 (0.0)

* Some animals received curative protocol: diminazene aceturate at 3.5 mg/kg and enrofloxacin at 7.5 mg/kg

For *B. bovis*, the frequency of infected male animals was 80.4% (37/46) at 20 days of age. At 2 months of age none became positive. At 2 and 4 months of age, the frequency of positive animals was 0 (0/10) and 30% (3/10) respectively. At 6 months, the infection rate was 80% (8/10), then 50% (5/10), 60% (6/10), and 50% (5/10) at 8, 9, and 14 months of age, respectively. Regarding *B. bigemina*, the infection frequency was 0.0% (0/10) at 2 and 4 months of age, and 80% (8/10), 70% (7/10), 60% (6/10), and 0.0% (0/10) at 6, 8, 9, and 14 months of age, respectively (Table 2).

In the dams of the female calves, 92.3% (48/52), 40.38% (21/52), and 5.7% (3/52) were infected with *A. marginale*,* B. bovis*, and *B. bigemina*, respectively. The infection rate of *A. marginale* in the female calves was 86.5% (45/52) at 20 days of age. It ranged from 30% (3/10) to 60% (6/10) between 2 and 6 months of age, and from 70% (7/10) to 80% (8/10) between 8 and 14 months. At 21, 22, and 25 months of age, the infection rate in females was 50% (5/10), 70% (7/10), and 60% (6/10), respectively (Table 3).

For *B. bovis*, the infection rate was 32.7% (17/52) in female calves at 20 days of age. Subsequently, at 2, 4, 6, 8, and 9 months of age, *B. bovis* was detected in 10% (1/10), 40% (4/10), 60% (6/10), 70% (7/10), and 40% (4/10) of the animals, respectively. On the following dates, the infection frequency was 100% at reproductive age (14 months), 80% (8/10) at 21 months, 40% (4/10) at 22 months, and 50% (5/10) at 25 months (Table 3).

Regarding *B. bigemina*, infection was detected in 51.9% (27/52) of the female calves at 20 days of age. Thereafter, the infection rates were 0.0% (0/10), 10% (1/10), 60% (6/10), 80% (8/10), 50% (5/10), 30% (3/10), 30% (3/10), 60% (6/10), and 50% (5/10) at 2, 4, 6, 8, 9, 14, 21, 22, and 25 months of age, respectively (Table 3). No TF agents were detected in F2 calves at birth.

### Infection dynamics of tick fever agents in F1 calves assessed by iELISA

**Fig. 3:**
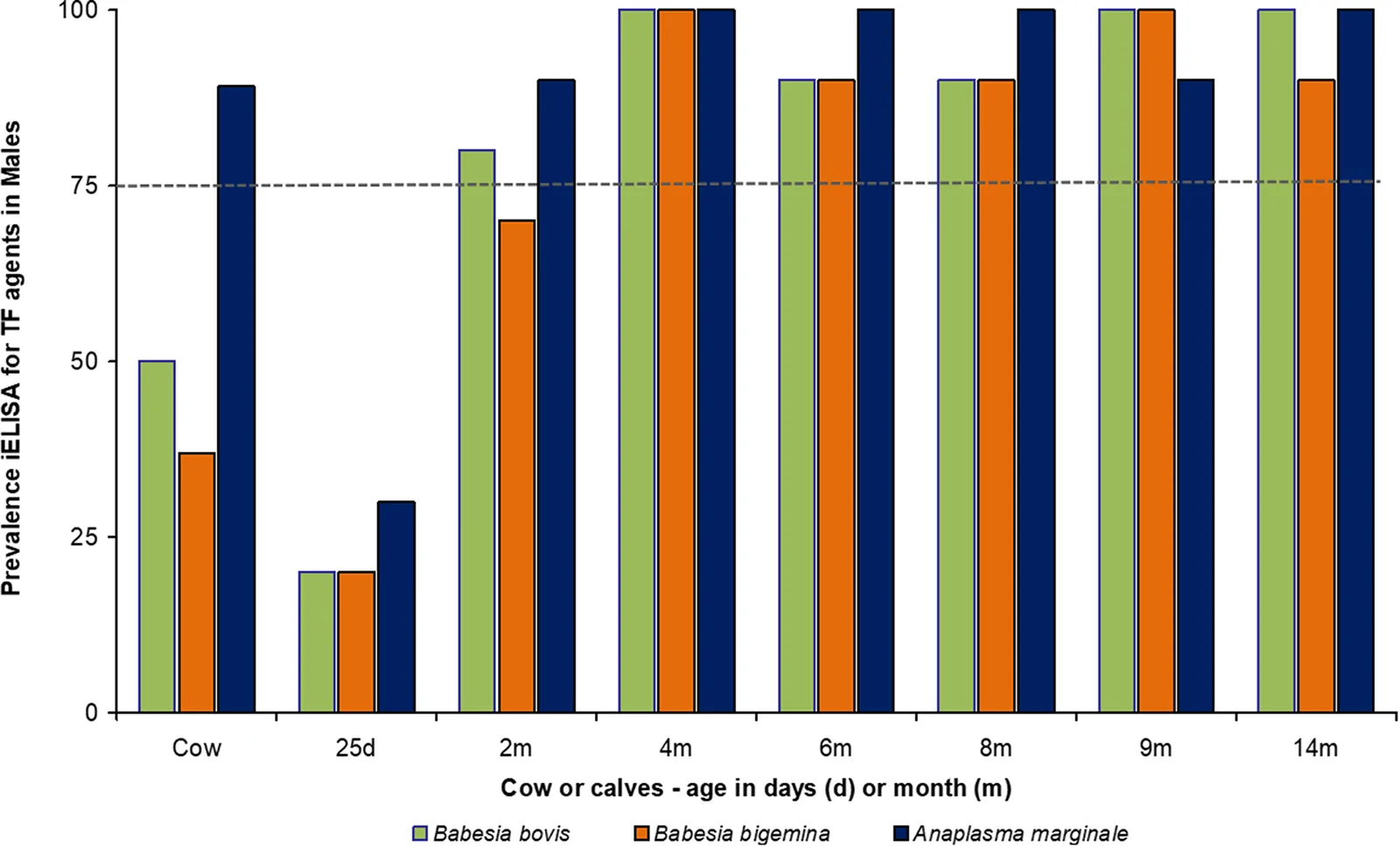
Number of animals (cows and male calves F1) positive for antibodies against *Babesia bovis*, *Babesia bigemina* and *Anaplasma marginale* detected by serology (iELISA)

In the dams of male calves, the prevalence of *A. marginale*, *B. bovis*, and *B. bigemina* was 89.1% (41/46), 50.0% (23/46), and 36.9% (17/46), respectively. For *B. bovis* and *A. marginale*, the prevalence remained above 75% from 2 to 14 months of age. A similar prevalence rate was observed for *B. bigemina* from 4 to 14 months of age (Fig. 3).

**Fig. 4:**
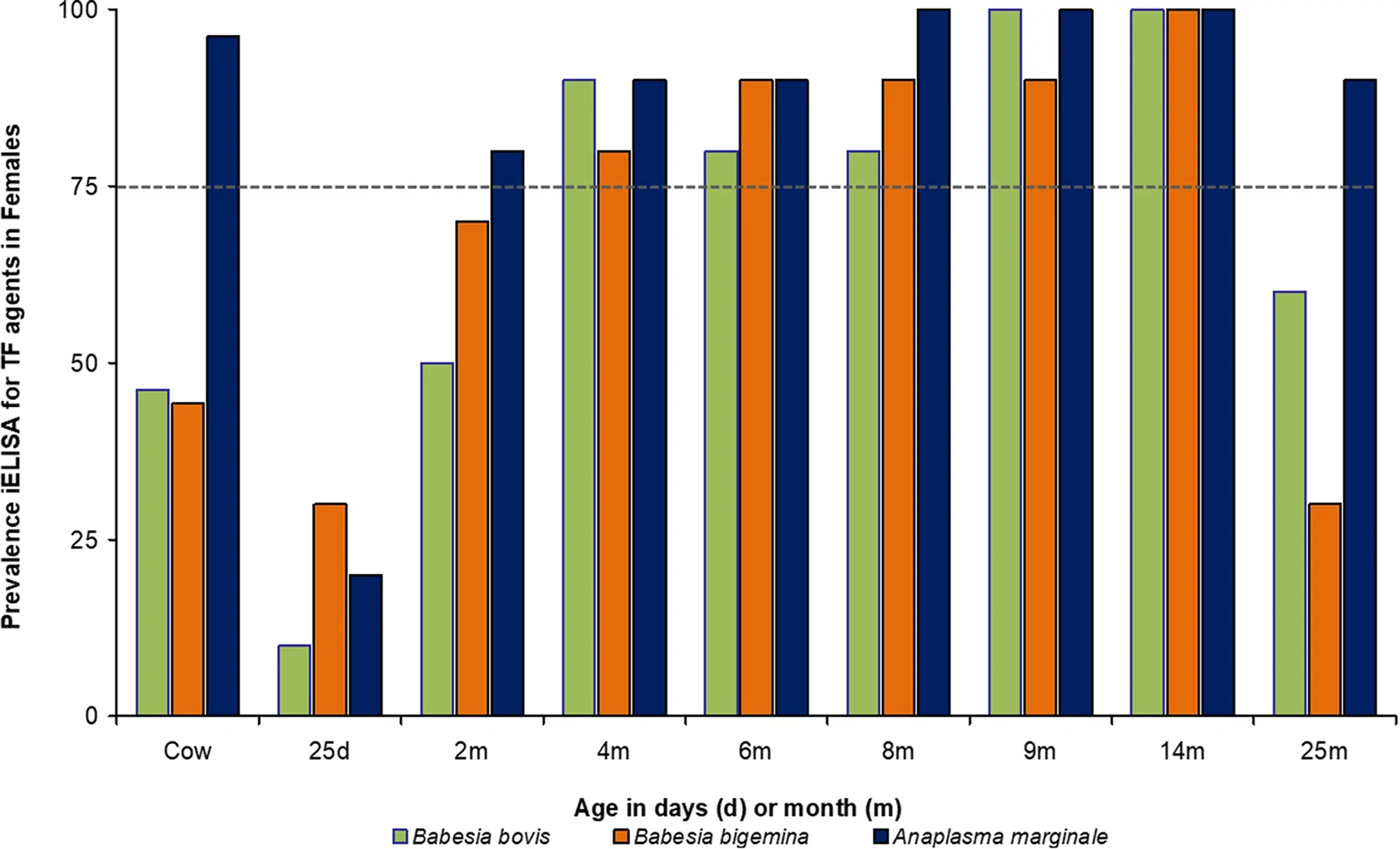
Number of animals (cows and female F1) positive for antibodies against *Babesia bovis*, *Babesia bigemina* and *Anaplasma marginale* detected by serology (iELISA)

In the dams of female calves, the prevalence of *A. marginale*, *B. bovis*, and *B. bigemina* was 96.2% (50/52), 46.2% (24/52), and 44.2% (23/52), respectively. In the female calves, *A. marginale* prevalence exceeded 75% after 2 months of age and remained at this level up to 25 months. For *B. bovis* and *B. bigemina*, the prevalence exceeded 75% from 4 to 14 months of age. At 25 months of age, the seroprevalence by iELISA for these agents was 60% (6/10) and 30% (3/10), respectively (Fig. 4).

Antibody titers against *A. marginale* were significantly higher (*p* ≤ 0.05) in male calves compared to female calves at 4 and 9 months of age. Regarding antibody titers against *B. bovis* and *B. bigemina*, male calves also presented higher levels compared to females between 6 and 9 months of age (Table 4).

**Table 4:** iELISA antibody titers against tick fever agents in male and female beef calves

Day (d) or month (m)	TF agents	Sex / Means ± Standard Deviation*										Pr > Chi-Square
		Female					Male					
20d	*A. marginale*	0.003	±	0.004	A	d	0.002	±	0.003	A	b	0.6274
2 m		0.07	±	0.08	A	c	0.16	±	0.14	A	a	0.0954
4 m		0.10	±	0.09	B	abc	0.29	±	0.23	A	a	0.0113
6 m		0.16	±	0.10	A	abc	0.21	±	0.03	A	a	0.3256
8 m		0.11	±	0.05	A	bc	0.16	±	0.08	A	a	0.1300
9 m		0.18	±	0.07	B	a	0.26	±	0.14	A	a	0.0143
14 m**		-					0.24	±	0.10		a	
25 m***		0.10	±	0.05		bc	-					
Pr > Chi-Square		0.00					0.1673					
20d	*B. bovis*	0.004	±	0.007	A	c	0.0004	±	0.001	A	c	0.7465
2 m		0.03	±	0.04	A	c	0.08	±	0.09	A	b	0.1888
4 m		0.08	±	0.08	A	ab	0.12	±	0.11	A	ab	0.3436
6 m		0.04	±	0.04	B	b	0.07	±	0.05	A	b	0.0373
8 m		0.06	±	0.05	B	a	0.16	±	0.07	A	ab	0.0032
9 m		0.12	±	0.09	B	a	0.27	±	0.19	A	a	0.0055
14 m**		-					0.20	±	0.10		a	
25 m***		0.02	±	0.03		b	-					
Pr > Chi-Square		< 0.0001					0.0010					
20d	*B. bigemina*	0.002	±	0.002	A	c	0.004	±	0.006	A	d	0.8467
2 m		0.05	±	0.08	A	bc	0.10	±	0.14	A	c	0.6454
4 m		0.11	±	0.08	A	ab	0.20	±	0.15	A	bc	0.1986
6 m		0.06	±	0.05	B	ab	0.19	±	0.11	A	bc	0.0091
8 m		0.07	±	0.06	B	ab	0.32	±	0.12	A	ab	0.0022
9 m		0.15	±	0.11	B	a	0.31	±	0.18	A	ab	0.0065
14 m**		-					0.30	±	0.12		ab	
25 m***		0.00	±	0.00		c	-					
Pr > Chi-Square		< 0.0001					0.0055					

^*^Mean values followed by the same letter (uppercase in the row and lowercase in the column) do not differ significantly from each other (*p* > 0.05)

^**^ Only males

^***^Only females

### Multivariable analysis of factors associated with *Anaplasma marginale* infection

**Fig. 5:**
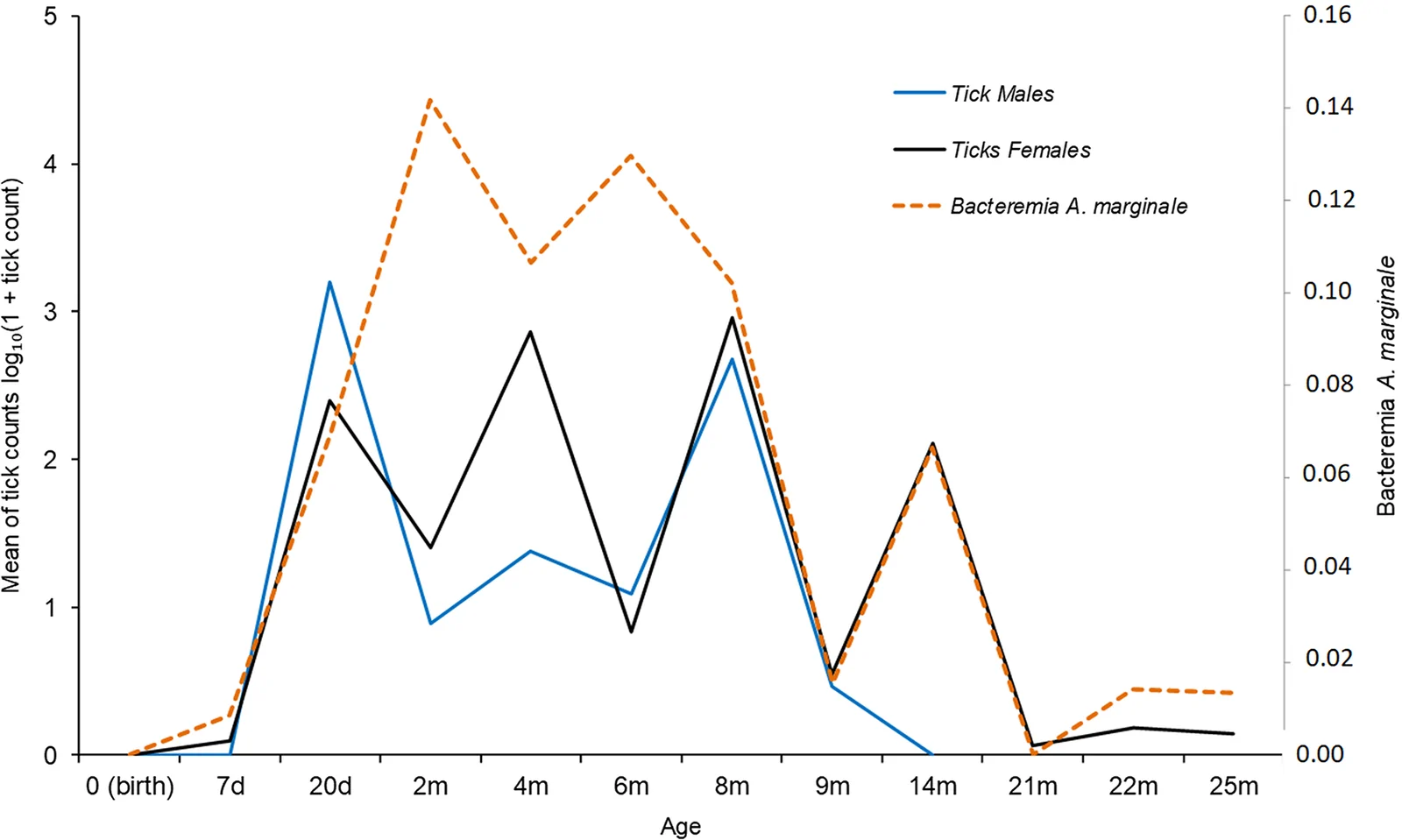
Tick counts and bacteremia by *A. marginale* in calves F1 during the study

Multivariable regression analyses identified tick count as the only variable significantly associated with *A. marginale* infection. The complete results of the multivariable regression analyses are presented in Table 5, while Fig. 5 provides a graphical illustration of tick burden and *A. marginale* bacteremia. In the logistic regression model, increasing tick burden was associated with higher odds of blood smear positivity (OR = 8.233; 95% CI: 4.096–16.548; *p* < 0.0001), whereas sex, therapeutic treatment against tick fever agents, packed cell volume, and total plasma protein concentration were not significantly associated with blood smear positivity (*p* > 0.05 for all variables).

**Table 5:** Results of the multivariable regression models evaluating factors associated with blood smear positivity and bacteremia of *Anaplasma marginale* in beef calves

Outcome	Predictor	Measure	Estimate	IC95%	Value of *p*
Blood smear positive for *A. marginale*	Female versus male	Odds ration	1.668	0.991–2.808	0.0540
	Tick coutn = log₁₀(1 + Tick count)*		8.233	4.096–16.548	< 0.0001
	Treatment for TF agents		0.949	0.115–7.860	0.9614
	Packed cell volume		1.020	0.954–1.091	0.5530
	Total plasmatic protein		1.181	0.829–1.682	0.3569
	Presence of *H. irritans*		Not estimable**		
	Presence of *S. calcitrans*		Not estimable**		
	Presence of Tabanidae		Not estimable**		
Bacteremia value	Female versus male	Beta coefficient	0.017	−0.031–0.066	0.4846
	Tick coutn = log₁₀(1 + Tick count)*		0.145	0.073–0.218	< 0.0001
	Treatment for TF agents		−0.035	−0.150–0.080	0.5504
	Packed cell volume		−0.003	−0.012–0.005	0.4290
	Total plasmatic protein		0.008	−0.009–0.025	0.3416
	Presence of *H. irritans*		Not reported**		
	Presence of *S. calcitrans*		Not reported**		
	Presence of Tabanidae		Not reported**		

* Tick count was included in the model as log10(1 + tick count). Therefore, the OR of 8.233 refers to a one-unit increase in the log10-transformed variable and should not be interpreted as the effect of one additional tick. A one-unit increase corresponds to a 10-fold increase in (1 + tick count). For example, an animal with 19 ticks has 8.233-fold higher odds of being blood-smear positive for *A. marginale* than an animal with 1 tick.

**Not estimable = the model could not estimate the parameter because of complete or quasi-complete separation, absence of exposed observations, or insufficient information in the data. Not reported = the estimate was not presented because the number of exposed observations was too small to provide a reliable and biologically meaningful result

Likewise, in the multivariable linear regression model, tick count was the only variable significantly associated with bacteremia (β = 0.145; 95% CI: 0.073–0.218; *p* < 0.0001), whereas sex, therapeutic treatment against tick fever agents, packed cell volume, and total plasma protein concentration were not significantly associated with bacteremia (*p* > 0.05 for all variables). Hematophagous flies were rarely observed during the study period. Consequently, the effects of *H. irritans*, *S. calcitrans*, and Tabanidae on blood smear positivity and bacteremia could not be reliably estimated because of the extremely limited number of exposed observations (Table 5).

The low parasitemia levels observed for *Babesia bovis* and *B. bigemina* precluded any comparative or multivariable analyses involving these pathogens.

### Acaricide treatments and therapeutic interventions against tick fever agents during the experiment

About therapeutic treatments for TF, 10 curative treatments were administered to male calves. Of these, 30% (3/10) were performed at 20 days of age, 20% (2/10) at 2 months, 10% (1/10) at 6 months, and 40% (4/10) at 9 months of age—approximately 45 days after the onset of feedlot confinement. Diagnosis via blood smear confirmed the presence of *A. marginale*, with bacteremia levels ranging from 0.8% to 1.6%.

In female calves, nine curative treatments were carried out. Of these, 11.1% (1/9) occurred at 20 days, 11.1% (1/9) at 2 months, 11.1% (1/9) at 3 months, 11.1% (1/9) at 4 months, 22.2% (2/9) also at 5 months, 11.1% (1/9) at 6 months, and 22.2% (2/9) at 13–14 months of age. Diagnosis by blood smear confirmed *A. marginale* infection in these animals, with bacteremia levels ranging from 0.4% to 1.1%.

Due to the occurrence of clinical cases of tick fever in over 40% of calves at 20 days of age—later confirmed as anaplasmosis—all animals received prophylactic treatment with imidocarb dipropionate on that date.

In total, male and female calves received six and nine acaricide treatments, respectively. Both sexes were treated at 20 days of age, and subsequently at 2, 4, 6, and 8 months. In addition to these, female calves received further treatments at 14, 20, and 22 months of age.

## Discussion

This study provides relevant information on the vertical transmission rate, infection dynamics, and potential risk factors associated with bovine tick fever in beef calves raised in a tropical climate. The findings showed a higher clinical occurrence of *A. marginale* compared to *B. bovis* and *B. bigemina* in the calves, a trend increasingly reported in pasture-based cattle systems in tropical regions (Souza et al. 2021; Heller et al. 2022; Cavalcante et al. 2025). A possible explanation for this trend may be related to the frequent use of synthetic chemical products for tick control, which, in turn, may reduce the frequency of *Babesia* spp. infections in cattle (Johnston et al. 1981). *Babesia* spp. infection in the herd is more dependent on the presence of cattle ticks; therefore, strategic applications of acaricide treatments reduce the tick population and, consequently, the prevalence of *Babesia* spp. in the herd. This hypothesis was also raised by Zapa et al. (2024), who attributed the low frequency of *B. bovis* observed in their study to this same mechanism.

In the present study, vertical transmission was observed only for *A. marginale*, with a rate of 6.5%. According to the literature, infection rates for this rickettsia in calves range from 6.7% in beef cattle (Grau et al. 2013) to 20–25% in dairy cattle (Costa et al. 2016). Recently, Leal et al. (2024) reported clinical anaplasmosis in 20-day-old calves on the same farm studied here, suggesting vertical transmission as a possible cause. However, the current findings do not support the hypothesis proposed by Leal et al. (2024). While *A. marginale* infection was detected in 6.5% of calves at birth to 7 days old, prevalence rose sharply to 84–86% by 20 days of age, indicating a strong external transmission factor. Three possible routes of infection at this stage are proposed: (i) iatrogenic transmission, (ii) blood-feeding insects, and (iii) cattle ticks.

The first proposed route can be reasonably excluded because sterile needles and syringes were used for each animal throughout the study, excluding the possibility of iatrogenic transmission. Consequently, arthropod vectors remained the most plausible sources of infection. Studies investigating the transmission of *A. marginale* by hematophagous flies have produced inconsistent results, with successful transmission being demonstrated only under specific forced experimental conditions (Sanders 1933; Potgieter et al. 1981), whereas several other studies failed to demonstrate transmission by these insects (CSIR – The Council of Scientific and Industrial Research, 1934; Taylor 1935; Mackerras et al. 1942; Peterson et al. 1977; Scoles et al. 2005, 2008; Heller et al. 2025). Likewise, long-term field studies involving *S. calcitrans* and *H. irritans* have suggested that these flies are unlikely to play a major epidemiological role in the transmission of *A. marginale* under natural conditions (Peterson et al. 1977; De Moraes et al. 2024). In the present study, no hematophagous flies were observed during the first 20 days of life, corresponding to the period in which most primary infections were presumed to have occurred. Thereafter, observations of *H. irritans* and *S. calcitrans* were rare on the animals after 20 days of age, precluding a reliable statistical assessment of their potential association with *A. marginale* infection. Therefore, the present study provides no evidence supporting a measurable epidemiological role of hematophagous flies in the transmission of *A. marginale* under the conditions evaluated.

The third hypothesis considers cattle ticks as a potential epidemiological source of *A. marginale* infection. Evidence of tick infestation was observed shortly after birth, as indicated by the presence of engorged *R. microplus* females (4.5–8 mm) on calves at 20 days of age, consistent with ticks approaching detachment after a complete feeding cycle (Wharton and Utech 1970). This observation indicates that tick infestation occurred during the first days of life. Furthermore, multivariable analyses demonstrated that tick burden was the only variable independently associated with both blood smear positivity and bacteremia after adjustment for age, sex, therapeutic treatment, packed cell volume, and total plasma protein concentration. Although these findings do not demonstrate that *R. microplus* was the primary vector responsible for *A. marginale* transmission, they provide observational evidence consistent with a potential epidemiological role of cattle ticks in the natural transmission of *A. marginale* under the field conditions evaluated in the present study. Therefore, the results support, but do not prove, the hypothesis that early exposure to *R. microplus* may have contributed to the occurrence of primary *A. marginale* infections observed in calves during the first weeks of life.

An emerging concern is the increasing occurrence of clinical anaplasmosis in calves as young as 20 days old, an unusual pattern compared to past decades when maternal antibodies typically protected calves until 1–2 months of age (Hall 1960, 1963; Hal et al. 1968). In the present study, colostrum intake evaluated three days after birth was considered above the ideal level (McGuirre, 2003) for male (± 7.3; 6.2–8.9) and female (± 7.7; 6.2–9.2) calves; however, this was not sufficient to prevent clinical disease during the primary *A. marginale* infection observed at 20 days of age. Historically, a herd is considered enzootically stable for *Babesia* spp. when over 75% of animals are seropositive (Mahoney and Roos, 1972), a concept used for *A. marginale* by Lagranha et al. (2024). Although this study detected a high frequency of *A. marginale* infection by qPCR at 20 days of age, these molecular findings should be interpreted as evidence of active infection at the time of sampling rather than as indicators of long-term herd immunity. Likewise, seroprevalence exceeding 75% until eight months of age demonstrated widespread previous exposure to *A. marginale*, whereas the occurrence of clinical anaplasmosis represented a distinct biological outcome that should be interpreted separately from molecular detection and serological evidence of previous exposure. Clinical cases were observed in males at 8–9 months of age (35–45 days after feedlot entry) and in females at approximately 13–14 months of age. Outbreaks of anaplasmosis in recently feedlot-adapted males after 30–60 days have also been reported in other regions and are frequently associated with immunological stress (Leal et al. 2024). Likewise, the occurrence of clinical cases in 13–14-month-old heifers may have been influenced by the physiological changes associated with the onset of estrus (Subandrio and Noakes 1997). A similar phenomenon has also been described for *Trypanosoma vivax* (De Melo Junior et al. 2024). Comparable observations have been reported in dairy cattle raised under tropical conditions, where more than 75% of calves were seropositive by four months of age (Cavalcante et al. 2025), yet animals continued to experience recurrent clinical and parasitological relapses despite previous exposure. Similar patterns have also been documented in other tropical regions (Souza et al. 2021; Heller et al. 2022). Collectively, these findings highlight that molecular detection, serological evidence of previous exposure, and clinical disease represent distinct biological outcomes and should therefore be interpreted separately when assessing the epidemiology of *A. marginale* infection.

For this reason, it is important to recognize that seropositivity alone does not necessarily reflect protective immunity against *A. marginale*. Although serological assays provide valuable information regarding previous exposure to the pathogen, protection against clinical anaplasmosis depends on a complex interaction between humoral and cell-mediated immune responses, as well as pathogen- and host-related factors. The magnitude of the infective dose, repeated exposure to infected ticks, and host genetic background may all influence the development and maintenance of protective immunity. Therefore, although serological prevalence remains an important epidemiological indicator, it should not be interpreted as the sole determinant of protective immunity against clinical anaplasmosis.

It is important to clarify that this study does not aim to challenge the established criteria for enzootic stability proposed by Mahoney and Roos (Mahoney and Ross 1972) for *Babesia* spp., nor their later validation for *A. marginale* by Lagranha et al. (2024). These studies have made valuable contributions to our understanding of disease dynamics. However, some key differences may help explain the divergent findings observed here. Notably, while those studies were conducted in subtropical regions, the present research was carried out in a tropical environment. Significant differences in bacteremia levels have also been reported between these regions. In subtropical areas, *A. marginale* infection levels detected via blood smear are typically below 0.02% (Aguirre et al. 1988; Morel et al. 2024). In contrast, animals in tropical regions often show bacteremia levels up to 15% (Heller et al. 2022; Zapa et al. 2024), with outbreaks reaching as high as 32.4% (Leal et al. 2024).

Another factor that may help explain the occurrence of clinical anaplasmosis at various ages, despite the herd reaching serological “stability” for tick fever, is the influence of temporal changes. Since 1972, significant shifts have occurred in cattle genetics and management. Advances in reproductive technologies and genetic selection have led to modern animals with much higher productive potential. However, many of these cattle, particularly European-derived breeds introduced into tropical regions, lack historical exposure to ticks and tick fever agents, limiting natural selection for disease tolerance. Additionally, climate change has impacted *R. microplus* population dynamics. In the study region, this tick species now completes up to five generations per year (Nicaretta et al. 2021; De Aquino et al. 2024), compared to a maximum of four decades ago (Carneiro et al. 1992). Intensified production systems have also led to much higher stocking rates, from 0.21 AU/ha in the past (Sereno et al. 2000) to as high as 6.9 AU/ha today (Difante et al. 2010), increasing tick pressure significantly. Consequently, animals genetically superior for productivity tend to be more susceptible to tick infestations (De Melo Junior et al. 2023) and more vulnerable to TF infections (De Moraes et al. 2024).

Some studies have proposed that *A. marginale* genetic diversity may influence the occurrence of clinical disease within herds (Machado et al. 2015; Hove et al. 2018). However, this association was not observed in other studies, including research conducted on the same property as the present work (Leal et al. 2024; Cavalcante et al. 2025). Additionally, since this is a closed herd with no introduction of outside animals, the previously discussed factors appear to offer more plausible explanations for the clinical cases observed. Although male calves showed higher antibody titers at specific time points in this study, no difference in clinical disease incidence between sexes was found. It is also important to highlight that serological titers do not necessarily reflect protective immunity (Noh et al. 2010; Albarrak et al. 2012).

Regarding the acaricide treatments administered to the animals, treatment intervals before weaning were 33, 76, 59, and 68 days, while post-weaning heifers had intervals of 184, 195, and 40 days. This likely reflects the development of acquired resistance to ticks through repeated exposure. Supporting this, De Melo-Junior et al. (2023) found significantly higher tick burdens in naïve calves compared to older animals with prior infestations. The reduced tick challenge observed in heifers between 9 and 25 months of age in the present study contributed to a decrease in the prevalence of *B. bovis* and *B. bigemina* to 60% and 30%, respectively, along with lower antibody titers. However, no clinical cases of babesiosis were observed, consistent with findings by Cavalcante et al. (2025).

An important aspect that should be considered when interpreting the molecular findings is the influence of the preventive and therapeutic treatments administered during the study. Prophylactic treatment with imidocarb and subsequent therapeutic interventions with diminazene and enrofloxacin were necessary for animal welfare and were implemented after the occurrence of clinical cases. These treatments were likely to reduce pathogen burden, thereby decreasing the probability of detecting circulating pathogens by qPCR during subsequent sampling. This potential effect is particularly relevant for *Babesia* spp., since both imidocarb and diminazene are highly effective against bovine babesiosis and may substantially reduce or eliminate circulating parasites following treatment (Heller et al. 2022; De Aquino et al. 2025). In contrast, although these drugs also reduce *A. marginale* bacteremia and improve clinical recovery, they generally do not eliminate the infection completely, allowing many animals to remain persistently infected despite reduced bacterial loads (Facury-Filho et al. 2012; Alberton et al. 2015; Heller et al. 2022). Consequently, the molecular results presented in this study should be interpreted primarily as complementary information on infection dynamics rather than as direct estimates of pathogen prevalence after treatment. For this reason, the serological results were considered the most appropriate indicator for discussing pathogen prevalence and enzootic stability, as antibody responses are substantially less affected by therapeutic interventions than molecular detection.

The findings of the present study provide important epidemiological information that may contribute to improving the understanding of bovine tick fever under tropical production systems. The results suggest changes in the infection dynamics, the age at which primary and subsequent clinical cases of anaplasmosis occur, and the interpretation of enzootic stability under these conditions. Although the observed associations should not be interpreted as evidence of causality, they highlight important epidemiological patterns that deserve further investigation and should be considered by researchers, veterinarians, and cattle producers when developing surveillance and control strategies for bovine tick fever.

## Conclusion

Among pasture-raised beef calves in tropical regions exposed to * R. microplus*, qPCR detected *A. marginale* DNA in approximately 85% of calves by 20 days of age, whereas *B. bovis* and *B. bigemina* DNA were detected in 60–80% of animals at the same age, demonstrating that active infection with tick fever agents occurred very early in life. Serological analyses showed that more than 75% of animals had been exposed to tick fever agents by 2–4 months of age. However, previous exposure, as reflected by seropositivity, was not consistently associated with protection against clinical anaplasmosis, since clinical cases requiring therapeutic intervention occurred in male calves at 20 days of age and again at 8–9 months of age, as well as in female calves at 20 days of age and in heifers at 13–14 months of age. These findings indicate that molecular detection, serological evidence of previous exposure, and the occurrence of clinical disease represent distinct biological outcomes that should be interpreted separately when evaluating the epidemiology of *A. marginale* under tropical conditions.

Finally, conclusions regarding *B. bovis* and *B. bigemina* should be interpreted with caution because the consistently low occurrence and parasitemia of these pathogens throughout the study precluded more detailed statistical analyses and limited the epidemiological conclusions that could be drawn. Furthermore, a low molecular or serological prevalence of these protozoa in adult cattle should not necessarily be interpreted as evidence of reduced herd immunity during adulthood, as these diagnostic methods reflect active infection or previous exposure rather than the persistence of protective immunity. The findings of the present study, together with recent field observations, suggest that the epidemiology of *Babesia* spp. under tropical conditions may differ from the patterns classically described decades ago. Therefore, additional longitudinal studies are warranted to better characterize the current epidemiology of bovine babesiosis and its relationship with molecular and serological findings and long-term protective immunity.

## Data Availability

The datasets generated during and/or analyzed in the current study are available from the corresponding author upon reasonable request.
